# Bone temperature during cementation with a heatsink: a bovine model pilot study

**DOI:** 10.1186/1756-0500-7-494

**Published:** 2014-08-07

**Authors:** Edward Spurrier, Olivia Payton, Mark Latimer

**Affiliations:** 1 Peterborough City Hospital, Bretton Gate, Peterborough PE3 9GZ, UK; 2 35b Marryat Square, Wyfold Road, London SW6 6UA, UK

**Keywords:** Arthroplasty, Bone cements, Osteonecrosis, Heatsink

## Abstract

**Background:**

Bone cement is an effective means of supporting implants, but reaches high temperatures while undergoing polymerisation. Bone has been shown to be sensitive to thermal injury with osteonecrosis reported after one minute at 47°C. Necrosis during cementing may lead to loosening of the prosthesis. Some surgeons fill the joint cavity with cool irrigation fluid to provide a heatsink during cementing, but this has not been supported by research. This paper assesses a simple technique to investigate the efficacy of this method.

**Findings:**

We used a model acetabulum in a bovine humerus to allow measurement of bone temperatures in cementing. Models were prepared with a 50 mm diameter acetabulum and three temperature probe holes; two as close as possible to the acetabular margin at half the depth of the acetabulum and at the full depth of the acetabulum, and one 10 mm from the acetabular rim. Four warmed models were cemented with Palacos RG using a standard mixing system and a 10 mm polyethylene disc to represent an acetabular component. Two of the acetabular models were filled with room temperature water to provide a heatsink. An electronic probe measured temperature at 5 second intervals from the moment of cementing.

In the models with no heatsink, peak temperature was 40.3°C. The mean temperature rise was 10.9°C. In the models with a heatsink, there was an average fall in the bone temperature during cementing of 4.4°C.

**Conclusions:**

These results suggest that using a heatsink while cementing prostheses may reduce the peak bone temperature. This study demonstrates a simple, repeatable technique which may be useful for larger trials.

## Findings

Polymethylmethacrylate (PMMA) bone cement is an effective means of supporting prostheses in bone. However, it reaches high temperatures during polymerisation, and it might be possible for the temperature of bone cement to heat bone enough to cause osteonecrosis at the bone-cement interface. This might be associated with a poorer mechanical structure and an increased chance of loosening [1-3]. Some surgeons routinely use room-temperature saline as a heatsink during cementing as a means of trying to keep the bone temperature down, either by filling the joint cavity with saline or by constant irrigation, once implantation is complete. This has not been supported by research and therefore we have performed a short *in-vitro* pilot study to assess a technique for investigating whether using saline as a heatsink does in fact lead to lower bone temperatures during cement polymerisation.

Thermal bone injury has been shown to lead to significantly lower osteocyte viability. This may weaken the fixation between bone and cement and thus lead to aseptic loosening [1]. One paper proposes that migration of the femoral prosthesis is caused by resorption of osteonecrotic bone following thermal injury [2].

There is little evidence to show the precise temperature at which osteonecrosis occurs. A bone temperature of 47°C for 1 minute has been shown to cause osteonecrosis [4]. Bone temperatures exceeding 88.8°C have been recorded when cementing hip resurfacing components in an experimental model [5], and injection of PMMA cement in to rabbit lumbar vertebrae has been shown to produce focal bone necrosis [6]. It has also been found that cooling the femoral canal in a model before cementing a femoral stem led to lower bone-cement interface temperatures and higher shear strength of the stem:cement interface [7].

It has been shown that *in vivo* bone temperatures during hip resurfacing [8] can be reduced through copious lavage and the use of a suction catheter in the lesser trochanter. This reduced peak bone temperature from 68°C to 36°C. This is a slightly different technique to that we propose, in which the joint cavity is filled with saline.

This pilot study aims to demonstrate whether a fluid heatsink is likely to reduce the peak temperature of the bone seen during implant cementation.

## Methods

**Figure 1:**
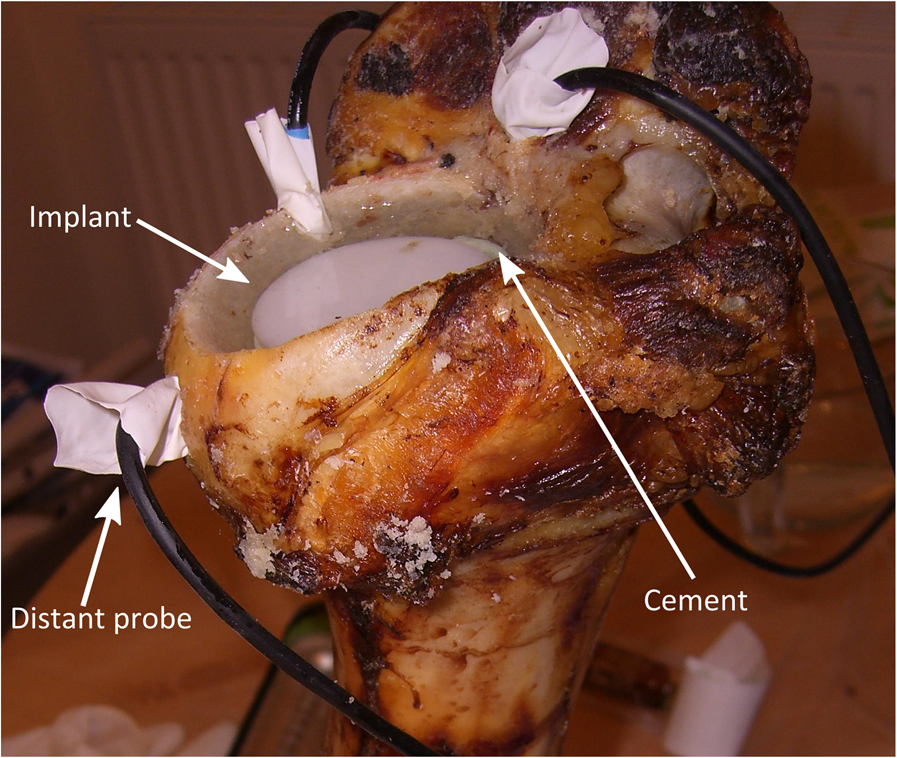
Wet bone model showing implant and probes during cementation.

The technique of filling a joint cavity with saline is applicable to cementing knee components as well as the acetabular and femoral components in hip arthroplasty. An acetabular model was chosen as it was felt it would be easiest to reproduce reliably.Four bovine humeri were obtained. Their proximal articular surfaces were drilled with a 50 mm hole saw to the maximum depth of the hole saw. A cylindrical hole was formed with an identical depth in each case to form a simple model acetabulum. Three temperature probe holes were drilled: one as close as possible to the acetabulum and to the same depth as the acetabulum; one as close as possible to the acetabulum and to half its depth; and one 10 mm away from the acetabulum and to half its depth. A 50 mm diameter, 10 mm thick disc of ultra-high molecular weight polyethylene (UMHWPE) was used to represent the acetabular bearing component. The experimental arrangement is shown at Figure 1.

Each model was heated to 38°C, monitoring the bone temperature with a simple temperature probe. Two models were cemented with no saline heatsink. A single mix of Palacos RG 20 bone cement (Heraeus, UK) was placed directly in the acetabulum and the UMHWPE disc used to pressurise. Temperature was monitored using a three probe temperature monitor and the temperature in each measurement hole was recorded every 5 seconds. Once a stable maximum temperature was reached, the experiment was stopped.

Two models were cemented with a water heatsink. The procedure was identical but as soon as the UMHWPE disc was inserted the acetabular cavity was filled with room temperature water. Since the bone was somewhat porous some of the first fill drained away and the acetabulum was refilled. 150 ml of water was used in each case. Again the temperature recordings were stopped once a stable maximum temperature was achieved.

Arithmetic means were calculated for the results from each prove across both bone models. A mean temperature for all three probes was also calculated.

## Results

**Figure 2:**
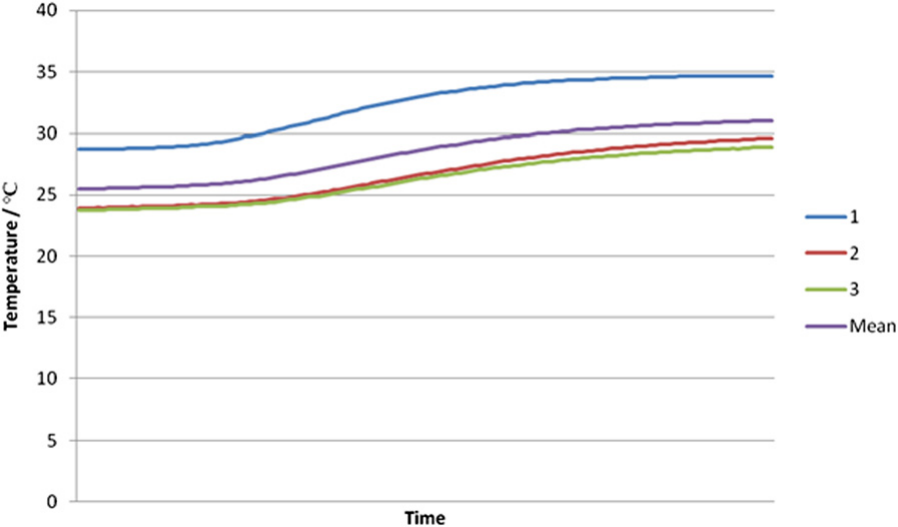
Temperature trend in dry bone models.

In the dry bone model the average starting temperature was 26.5°C, as some cooling took place before starting cementing. The peak temperature recorded was 40.3°C in the closest probe to the bone cement. The mean temperature after cementing was 36.4°C and the mean rise in temperature 10.9°C. These data are shown in Table 1. See Figure 2 for a graph showing the trend of temperature across both bone models.

**Table 1:** Mean temperatures (°C) between sensors for dry models

**Sensor:**	**1**	**2**	**3**	**Mean**
Start	28.7	23.9	23.8	25.5
Finish	40.3	33.6	35.4	36.4
Change	11.6	9.6	11.6	10.9

Sensor 1: close half depth; 2: close full depth; 3: distant.

**Figure 3:**
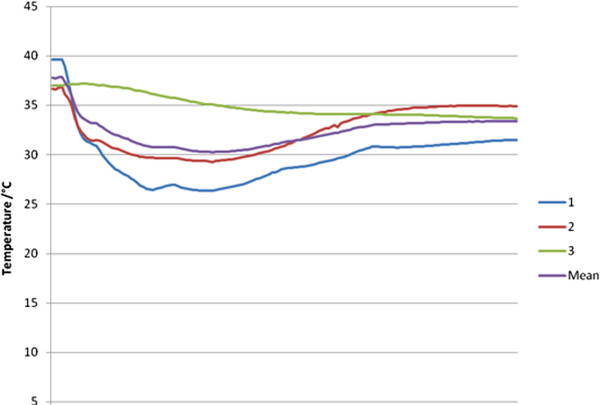
Temperature trend in wet bone models.

In the wet bone model, the average initial temperature was 37.8°C, as technique had been refined and there was a shorter delay before starting cementing. As soon as the water heatsink was applied there was a fall in temperature across all probes with the lowest temperature, 26.4°C, recorded at the probe closest to the acetabulum and at half its depth. The mean fall between starting temperature and minimum temperature was 30.2°C. The temperature then rose steadily but did not return to the starting temperature. The peak end of measurement temperature was 34.9°C recorded in the close, full-depth probe. The closest half-depth probe recorded a fall of 8°C and the mean change in temperature was a fall of 4.4°C. These data are shown in Table 2 and the trend can be seen in Figure 3. It is presumed that the effect of the heatsink is more marked closer to the acetabulum as there is an insulating effect from 10 mm of bone thickness.

**Table 2:** Mean temperatures (°C) between sensors for wet models

**Probe:**	**1**	**2**	**3**	**Mean**
Start	39.6	36.7	37	37.8
Low	26.4	29.2	33.7	30.2
End	31.6	34.9	33.7	33.4
Change	−8.0	−1.8	−3.3	−4.4

Sensor 1: close half depth; 2: close full depth; 3: distant.

## Discussion

This study technique was straightforward and repeatable, and might therefore be useful in a larger investigation of this subject. The trend of a significant temperature fall when a heatsink is used in this study suggests that filling the joint cavity with irrigation fluid is likely to keep bone temperatures down during cementing. This may therefore reduce the risk of later component loosening. These findings support the slightly different technique used by Gill et al. [8] and we therefore propose that irrigation fluid should be used to provide a heatsink during component cementing.

This small *in vitro* study has some significant limitations. It is not possible to model the effect of intraosseous circulation, which may have a cooling effect as the cement temperature rises, although one canine [9] model showed that interrupting circulation with a tourniquet did not change the peak cement temperature compared to limbs with normal blood flow. The losses from the system in to the environment are likely to be higher due to the lower total mass and lack of surrounding soft tissues. It was not felt appropriate to perform analysis of statistical significance with only two models in each group.

Irrigating the wound in hip and knee replacement surgery does not affect the compressive strength of the bone cement [10] so this technique should not itself impair the fixation of cemented implants.

Interestingly one study using ovine models found that thermonecrosis did not occur despite temperatures exceeding 49.3° [11] although this contradicts several other papers which suggest that thermally induced bone injury may be significant [5,6,12].

One paper reports an inflammatory reaction with bone cement temperatures exceeding 70 degrees [12]. Although they did not find bone or marrow necrosis they did report fibrous scar tissue formation which may lead to early loosening and subsequent implant failure. Similar findings were reported by Krause et al., with formation of a mechanically unsound fibrous membrane at the cement-bone interface, which was significantly thicker in cases where histological evidence of osteonecrosis was seen [13]. A vertebroplasty study also showed a fibrotic reaction surrounding a zone of osteonecrosis [14]. Although osteolysis due to macrophage activity is the main cause of “cement disease” loosening [15], poor initial fixation may have an impact on implant longevity.

This pilot study therefore suggests that a fluid heatsink may reduce peak bone temperature during cementation and therefore reduce the risk of implant loosening due to osteonecrosis. Further research is required with an *in-vivo* replication of this work to demonstrate the effect in the living patient.

## Abbreviations

PMMA: Poly methyl methacrylate.

## Competing interests

Bone cement for this study was supplied by Heraeus UK. No other financial or other competing interests are declared.

## Authors’ contributions

ES and ML designed the study, carried out the experiments, and drafted the paper structure. OP completed the manuscript text and undertook the literature review. All authors read and approved the final manuscript.
